# Global discovery, expression pattern, and regulatory role of miRNA-like RNAs in *Ascosphaera apis* infecting the Asian honeybee larvae

**DOI:** 10.3389/fmicb.2025.1551625

**Published:** 2025-03-04

**Authors:** Xiaoyu Liu, Sihai Geng, Daoyou Ye, Wenhua Xu, Yidi Zheng, Fangji Wang, Jianpeng Lei, Ying Wu, Haibin Jiang, Ying Hu, Dafu Chen, Tizhen Yan, Rui Guo, Jianfeng Qiu

**Affiliations:** ^1^College of Bee Science and Biomedicine, Fujian Agriculture and Forestry University, Fuzhou, China; ^2^National and Local United Engineering Laboratory of Natural Biotoxin, Fuzhou, China; ^3^Apitherapy Research Institute of Fujian Province, Fuzhou, China; ^4^Apiculture Science Institute of Jilin Province, Jiling, China; ^5^Dongguan Maternal and Children Health Hospital, Dongguan, China

**Keywords:** chalkbrood, *Ascosphaera apis*, *Apis cerana*, milRNA, target mRNA, regulatory network

## Abstract

*Ascosphaera apis*, a specialized fungal pathogen, causes lethal infection in honeybee larvae. miRNA-like small RNAs (milRNAs) are fungal small non-coding RNAs similar to miRNAs, which have been shown to regulate fungal hyphal growth, spore formation, and pathogenesis. Based on the transcriptome data, differentially expressed miRNA-like RNAs (DEmilRNAs) in *A. apis* infecting the *Apis cerana cerana* worker 4-, 5-, and 6-day-old larvae (Aa-4, Aa-5, and Aa-6) were screened and subjected to trend analysis, followed by target prediction and annotation as well as investigation of regulatory networks, with a focus on sub-networks relative to MAPK signaling pathway, glycerolipid metabolism, superoxide dismutase, and enzymes related to chitin synthesis and degradation. A total of 606 milRNAs, with a length distribution ranging from 18 nt to 25 nt, were identified. The first nucleotide of these milRNAs presented a bias toward U, and the bias patterns across bases of milRNAs were similar in the aforementioned three groups. There were 253 milRNAs, of which 68 up-and 54 down-regulated milRNAs shared by these groups. Additionally, the expression and sequences of three milRNAs were validated by stem-loop RT-PCR and Sanger sequencing. Trend analysis indicated that 79 DEmilRNAs were classified into three significant profiles (Profile4, Profile6, and Profile7). Target mRNAs of DEmilRNAs in these three significant profiles were engaged in 42 GO terms such as localization, antioxidant activity, and nucleoid. These targets were also involved in 120 KEGG pathways including lysine biosynthesis, pyruvate metabolism, and biosynthesis of antibiotics. Further investigation suggested that DEmilRNA-targeted mRNAs were associated with the MAPK signaling pathway, glycerolipid metabolism, superoxide dismutase, and enzymes related to chitin synthesis and degradation. Moreover, the binding relationships between aap-milR10516-x and *ChsD* as well as between aap-milR-2478-y and *mkh*1 were confirmed utilizing a combination of dual-luciferase reporter gene assay and RT-qPCR. Our data not only provide new insights into the *A. apis* proliferation and invasion, but also lay a basis for illustrating the DEmilRNA-modulated mechanisms underlying the *A. apis* infection.

## 1 Introduction

*Ascosphaera apis* is a lethal fungal pathogen that exclusively invades honeybee larvae, giving rise to chalkbrood disease, which causes severe damage to bee health and sharp decline of colony population as well as productivity (Aronstein and Murray, 2010) *A. apis* spores are ingested orally with food by honeybee larvae and germinate at a low level in the lumen of the midgut, the septum between the midgut and the hindgut disappears at the pre-pupal stage, the spores enter the hindgut with the food residues and germinate violently upon the stimulation of oxygen, and the fungal mycelium rapidly develops and penetrate the intestinal wall and then the body wall, the mycelia pierce from the end of larvae and finally covered the entire larvae, generating a white or black chalkbrood mummy (Aronstein and Murray, 2010). *A. apis* has been verified to infect not only *Apis mellifera* larvae and bumblebee queens (Maxfield-Taylor et al., 2015), but also larvae of *Apis cerana* worker as well as drone.

In past decades, miRNAs have been discovered in diverse animals (Grimson et al., 2008), plants (Llave et al., 2002), and microorganisms (Chen et al., 2014; Nersisyan et al., 2020) and confirmed to play essential roles in numerous processes such as growth (DeVeale et al., 2021), development (Pei et al., 2023), metabolism (Agbu and Carthew, 2021), and immunity (Kim et al., 2017; Asgari, 2013). With the development of deep sequencing technology and relevant bioinformatics, a substantial amount of miRNA-like small RNAs (milRNAs) in fungi have been identified and documented (Lee et al., 2010; Huang and Evans, 2016; Lau et al., 2018). Previously, mixed samples of *A. apis* mycelium and spores were prepared followed by sequencing of corresponding cDNA libraries using small RNA-seq technology, a total of 118 milRNAs were predicted and found to play a potential regulatory part in the material and energy metabolism as well as MAPK signaling pathway (Guo et al., 2018). Accumulating evidence has demonstrated that milRNAs are engaged in the modulation of fungal mycelium growth, spore formation, and pathogenesis (Li et al., 2017; Lin et al., 2016; Guo et al., 2019). For instance, Villalobos-Escobedo et al. (2022) found that *Trichoderma atroviride* milRNAs could regulate hyphal regeneration by post-transcriptionally regulating cellular signaling processes involving phosphorylation events. Li et al. (2017) reported that mro-miR-33 promoted spore formation in *Metarhizium robertsii* through negative modulation of *brlA*, a key gene responsible for asexual spore production in filamentous fungi. Previous studies on pathogenic milRNAs were mainly associated with fungi infecting plants. Two milRNAs in *Rhizoctonia solani*, Rhi-milR-16, and Rhi-milR-92, were engaged in the degradation of host cell walls and antifungal compounds by regulating the expression of target genes relative to various pathogenic factors (Lin et al., 2016). Comparatively, due to a lack of relevant research, little is known about the role of milRNAs in the fungal infection of insects. Cui et al. (2019) discovered that bba-milR1, a miRNA-like RNA in *Beauveria bassiana*, could suppress host immunity by silencing the expression of the Toll receptor ligand Spätzle 4 (Spz4) gene in mosquitoes. Recently, Fan et al. (2024) identified 974 milRNAs in *A. apis* infecting *Apis mellifera ligustica* worker larvae, and found that DEmilRNAs included in four significant trends potentially modulated the expression of the target genes encoding secondary metabolite-associated enzymes, virulence factors, and MAPK signaling pathway.

*Apis cerana*, a bee species widely distributed in Asian countries such as China, Japan, and North Korea, is well-known for its long foraging period, ability to utilize scattered nectar sources, tolerance to low temperature, and strong disease resistance (Mi and Tan, 2007; Zhang et al., 2008; Qin et al., 2016), making it indispensable for maintaining ecosystem stability and promoting the apicultural development.

Following deep sequencing of cDNA libraries from *A. apis-and* un-inoculated gut tissues of *A. cerana* worker larvae annotated, we previously gained high-quality transcriptome datasets including both *A. cerana-and A. apis*-derived data. Here, trend analysis of DEmilRNAs in *A. apis* during the infection process was performed, followed by annotation of DEmilRNA-targeted mRNAs and in-depth investigation of the regulatory network associated with MAPK signaling pathway, glycerolipid metabolism, superoxide dismutase, and enzymes related to chitin synthesis and degradation, and further, the relationships between two DEmilRNAs and corresponding target mRNAs were verified using molecular approach. Our findings could not only provide novel insights into the *A. apis* proliferation and invasion, but also lay a foundation for clarifying the milRNA-mediated mechanisms underlying the *A. apis* infection of *A. cerana* larvae. Also, this work will offer candidates for functional dissection and promising targets for diagnosis and control of chalkbrood.

## 2 Materials and methods

### 2.1 Bee larvae and *Ascosphaera apis*

*Apis cerana* worker larvae were obtained from three colonies reared by the Host-Pathogen Interaction and Precision Medicine research team, College of Bee Science and Biomedicine, Fujian Agriculture and Forestry University (119.2369° E, 26.08279° N). The larvae were cultured at 35.0 ± 0.5°C and 90% relative humidity (RH), and fed with an artificial diet every 24 h. *A. apis* (Fuzhou strain) was isolated by our laboratory (Maxfield-Taylor et al., 2015) and conserved in China General Microbiological Culture Collection Center (NO. 40895).

### 2.2 Larval inoculation and gut sample preparation

Following the protocol developed by our lab, the *A. apis* spores were purified (Du et al., 2019; Guo et al., 2019a). The fresh spores were counted using a hemocytometer. In the treatment group, 3-day-old larvae (*n* = 3) were each fed 50 μL of the artificial diet containing spores with a final concentration of 1 × 10^7^ spores/mL. In the control group, 3-day-old larvae (*n* = 3) were each fed 50 μL of the artificial diet without spores. The larvae in these two groups were placed in two separate constant temperature and humidity chambers, and reared at 35.0 ± 0.5°C and 90% RH. After the larvae consumed the diet, a fresh diet without spores was added, thereafter fresh diet was added every 24 h. At 4, 5, and 6 day-old, 9 larvae in each group were dissected and the gut tissues were placed in RNA-Free Eppendorf (EP) tubes. The prepared gut samples were frozen in liquid nitrogen and stored at −80°C. The experiment was performed in triplicates.

### 2.3 RNA isolation, cDNA library construction, and deep sequencing

The sRNA-seq was prepared according to the established protocol, followed by total RNA isolation, cDNA library construction, sRNA-seq, and strict quality control of raw data (Chen et al., 2020). The gut tissues of *A. apis*-inoculated *A. c. cerana* worker 4-, 5-, and 6-day-old larvae (Aa-4, Aa-5, and Aa-6 group) were dissected and then subjected to total RNA isolation and cDNA library construction, followed by sRNA-seq and data quality control, there were three biological replicates of the *A. apis*-inoculated larval gut samples (Guo et al., 2019b; Xiong et al., 2020). The raw data are available in the NCBI SRA database under the BioProject number: PRJNA565611.

### 2.4 Quality control of raw data

Quality control was performed on the raw reads by: (1) filtering out reads with more than one base having a quality value below 20; (2) removing reads containing unknown bases (N); (3) filtering out 3′ or 5′ adapter sequences and discarding reads shorter than 18 bp; (4) filtering reads containing poly-A tails. The resulting clean reads were used for subsequent analysis. Clean reads were aligned against the GenBank database^1^ and the Rfam database^2^ using the Blastall tool, and reads aligning with rRNA, scRNA, snoRNA, snRNA, and tRNA were excluded.

### 2.5 Screening of *Ascosphaera apis* milRNAs

The clean tags were, respectively, aligned to the GenBank database,^3^ RFAM database,^4^ ribosome database,^5^ and *A. apis* reference genome (assembly AAP 1.0) by using the Blast tool to remove possible rRNA, scRNA, snoRNA, snRNA, tRNA, and those aligned clean tags to genomic exon, intron, and repeat regions (Xiong et al., 2020). Subsequently, the unaligned clean tags were compared with milRNA precursor sequences in the miRBase database^6^ to identify known milRNA sequences. Further, the unannotated tags were mapped to the *A. apis* reference genome (assembly AAP 1.0) using the Mireap_v0.2 software, and novel milRNA candidates were identified according to their genomic positions and hairpin structures. The TPM (tags per million) algorithm is utilized to normalize the expression level of all milRNAs. Furtherly, the length distribution and first nucleotide bias of milRNAs in the Aa-4, Aa-5, and Aa-6 groups were identified which were plotted using the GraphPad Prism 7 software.

### 2.6 Analysis of DEmilRNAs

First, the miRNA was analyzed differentially using the edgeR software with |log_2_(fold change)| > 1 and *p* < 0.05. Among them, the fold change in expression level between Aa-4 vs. Aa-5, Aa-4 vs. Aa-6, and Aa-5 vs. Aa-6 comparison groups were determined according to the following formula: (TPM in Aa-5)/(TPM in Aa-4) or (TPM in Aa-6)/(TPM in Aa-5) (Fan et al., 2024). Subsequently, Short Time-series Expression Miner (STEM) software (Ernst and Bar-Joseph, 2006) to analyze the trend of the total DEmilRNAs, with *p* < 0.05 as the significant threshold. The default parameters were as follows: The maximum unit change of the model profiles between time points was 1, the maximum number of output maps was 20, and the minimum fold change ratio of DEmilRNA was not less than 2.0. The results were exported from the STEM software and significant profiles were selected.

### 2.7 Prediction and annotation of target mRNAs of DEmilRNAs

Three software, RNAhybrid (v2.1.2) + svm_light (v6.01) (Enright et al., 2003), Miranda (v3.3a) (Krüger and Rehmsmeier, 2006), and Target Scan (Version:7.0) (Allen et al., 2005), were employed to predict the target mRNA of the DEmilRNAs, and the intersection of the three prediction results was regarded as a reliable set of target mRNAs. Subsequently screening obtained the targeted binding relationship with ΔG < −17 kcal/mol for subsequent analysis. Next, these target mRNAs were mapped to the GO^7^ and KEGG^8^ databases to gain corresponding annotations using the relevant tool in the Omicshare platform,^9^ with the default parameters. Finally, the different functional terms and KEGG pathways were presented through the Omicshare platform (see Footnote 9).

### 2.8 Construction of regulatory network for DEmilRNAs and target mRNA

In collaboration with the annotations in KEGG databases as well as relevant documentation (Asl et al., 2021), those target mRNAs associated with MAPK signaling pathway, virulence factor, and glycerolipid metabolism and corresponding DEmilRNAs were screened followed by construction of sub-networks, which were visualized by the Cytoscape v.3.2.1 software (Smoot et al., 2011).

### 2.9 Stem-loop RT-PCR and sanger sequencing

Randomly selected 3 milRNAs (milR-31-x, milR-7977-x, and milR-9993-y) for Stem-loop RT-PCR and Sanger sequencing. Then, specific Stem-loop primers and upstream primers (F) as well as universal downstream primers (R) were designed and synthesized by Sangon Biotech Co., Ltd. (Shanghai, China) (Supplementary Table S1). According to the method of Fan et al. (2024), total RNA was extracted using RNA extraction kit (Permoga, LS1040, China), followed by reverse transcription with Stem-loop primers. The resulting cDNA were used as templates for PCR amplification (Yeasen, 10102ES08, China). The products were then subjected to electrophoresis on a 1.5% agarose gel. Next, the target fragment was recovered, ligated with pMD-19 T vector (TaKaRa, 6,013, China), and transformed into *E. coli* DH5α (Tiangen, CB101-01, China). The single colony was transferred to the LB liquid medium to culture for 12 h, and a bit of bacterial liquid was then taken to conduct PCR confirmation. The bacterial liquid with a positive signal was sent to Sangon Biotech Co., Ltd. (Shanghai, China) for Sanger sequencing.

### 2.10 RT-qPCR

RT-qPCR detection was performed on a QuantStudio 3 fluorescent quantitative PCR system (ABI, Los Angeles, CA, United States), following the conditions: 95°C for 5 min; 95°C for 30s; 60°C for 30 s; 40 cycles.; melting curve program default system settings. The reaction system was as follows: 10 μL of SYBR Green Dye (Yeasen, 11202ES08, China), 1 μL each of upstream and downstream primers (2.5 μmol/L) (Table 1) as well as cDNA templates, and the DEPC treatment water was replenished to 20 μL. The *5.8S rRNA* gene (GeneBank accession number: NR_178140) was used as the internal reference. The relative expression level of each DEmilRNA and target mRNAs was calculated by using the 2^−ΔΔCt^ method (Ha and Kim, 2014). Each reaction was conducted with at least three samples in parallel and was repeated three times.

**Table 1:** Overview of the sRNA-seq data from *A. apis* infecting *A. cerana* worker larvae.

Group	Raw reads	Clean reads	Mapping ratio	Source
Aa-4	13119026.7	12537258.3 (95.56%)	7.66%	This study
Aa-5	11,905,316	11,494,486 (96.55%)	5.11%	This study
Aa-6	10,773,889	10,399,091 (96.52%)	6.43%	Xiong et al. (2020)

### 2.11 Dual luciferase analysis

The mimic and the negative control mimic (Mimic-NC) were commissioned to be synthesized by Shanghai Jimar Pharmaceutical Technology Company. The potential binding site sequences of milRNA and targeted genes were predicted by the software and cloned into the pmirGLO vectors. The mutant sequences of the above binding sites were designed and cloned into the pmirGLO vectors, and the bacterial fluids were sent to Sango Bioengineering (Shanghai) Co. for Sanger sequencing. The correctly sequenced bacterial fluids were transferred to fresh LB liquid medium. The plasmids were extracted using the Endotoxin Removal Plasmid Extraction Kit (Beijing AllStyle Gold Biotechnology Co., Ltd.).

HEK-293T cells were spread into 48-well cell culture plates and then placed into a 37°C incubator to continue incubation for 24 h, so that the cell density reached 90–95%. Cell transfection experiments were performed according to the instructions of Hieff Trans^®^ Liposomal Transfection Reagent (Yeasen, 40802ES02, China). Each group was transfected with 200 ng of plasmid and 20 pmol Mimics or Mimic-NC. After the transfection was completed and incubated for 24 h, the firefly fluoresceinase and kidney fluoresceinase were detected on a GloMax chemiluminescence detector using a dual-luciferase kit (Yeasen, 11402ES60, China), and the firefly fluoresceinase/kidney fluoresceinase ratio was calculated to obtain the relative expression folds. The experiment was repeated three times.

### 2.12 Statistical analysis

GraphPad Prism version 10 (GraphPad, San Diego, CA, United States) was used for graph construction and statistical analysis. Data are presented as the mean ± SD. Statistical analysis was performed using the one-way ANOVA or Two-way ANOVA, Tukey’s multiple comparisons (ns, *p* > 0.05; *, *p* < 0.05; **, *p* < 0.01; ***, *p* < 0.001).

## 3 Results

### 3.1 Quality control of sRNA-seq datasets

On average, 13119026.7, 11,905,316, and 10,773,889 raw reads were identified from the Aa-4, Aa-5, and Aa-6 groups, respectively. After quality control, the clean reads of Aa-4, Aa-5, and Aa-6 groups were 12537258.3, 11,494,486, and 10,399,091, respectively, accounting for 95.56, 96.55, and 96.52% of the raw reads. In addition, the mapping ratios were 7.66, 5.11, and 6.43%, respectively (Table 1).

### 3.2 Identification and structural analysis of *Ascosphaera apis* milRNAs during the infection process

**Figure 1:**
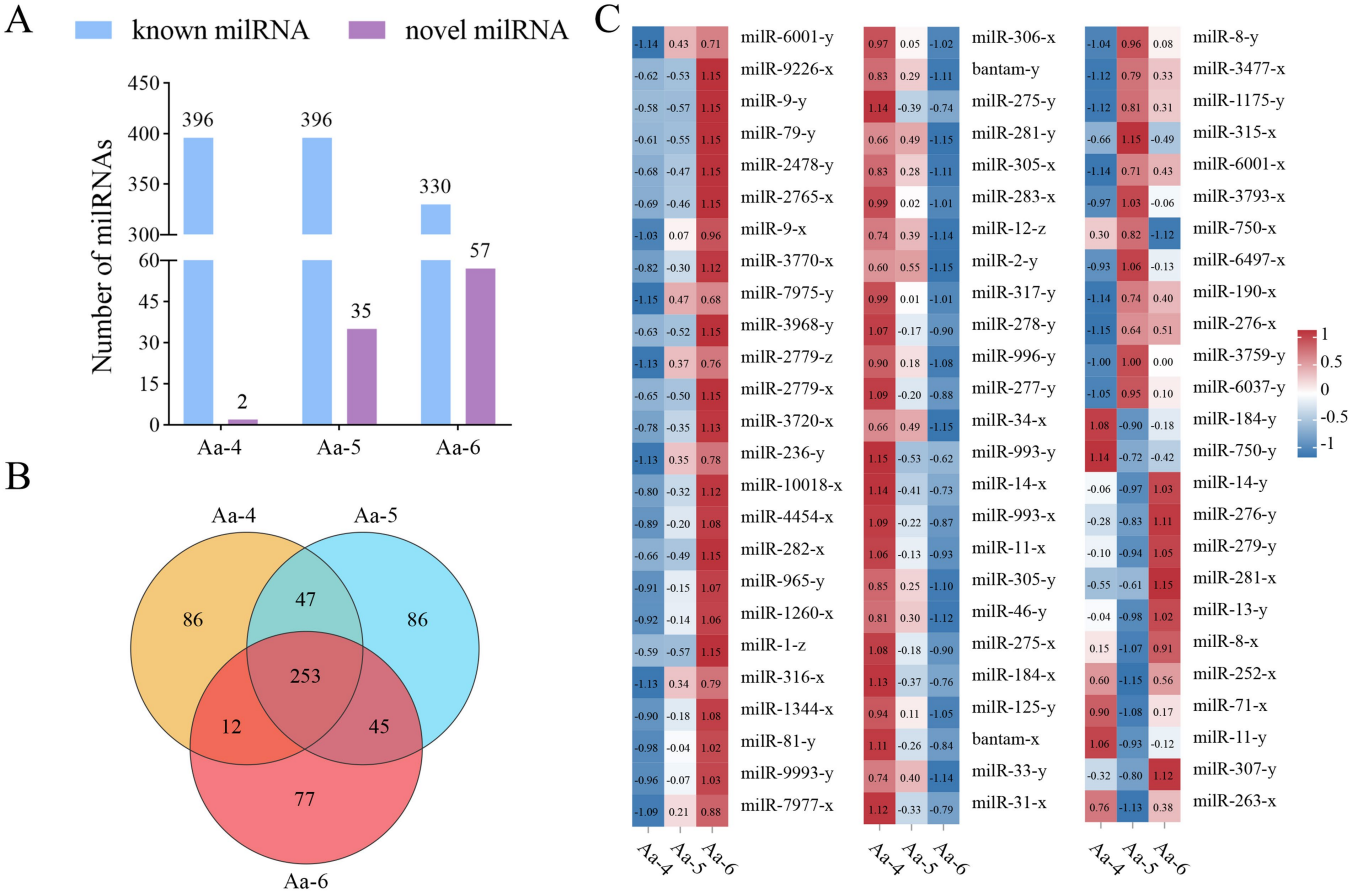
Number statistics, Venn diagram, and expression clustering of *A. apis* milRNAs. **(A)** Statistics of known and novel milRNAs in three different groups; **(B)** Venn diagram of milRNAs in three different groups; **(C)** Heat map of expression clustering of shared milRNAs by different groups.

In the Aa-4 group, 398 milRNAs were identified, including 396 known and 2 novel ones. A total of 431 milRNAs were detected in the Aa-5 group, including 396 known and 35 novel ones. Additionally, 387 milRNA, including 330 known and 57 novel milRNAs, were found in the Aa-6 group (Figure 1A). Venn analysis indicated that 253 milRNAs were shared by the Aa-4, Aa-5, and Aa-6 groups, whereas there were 86, 86, and 77 specific milRNAs, respectively (Figure 1B). As shown in Figure 1C, these shared milRNAs displayed various expression trends during the process of *A. apis* infection, with 68 (54) milRNAs showing a continuous up-regulation (down-regulation) trend (Supplementary Figure S1).

**Figure 2:**
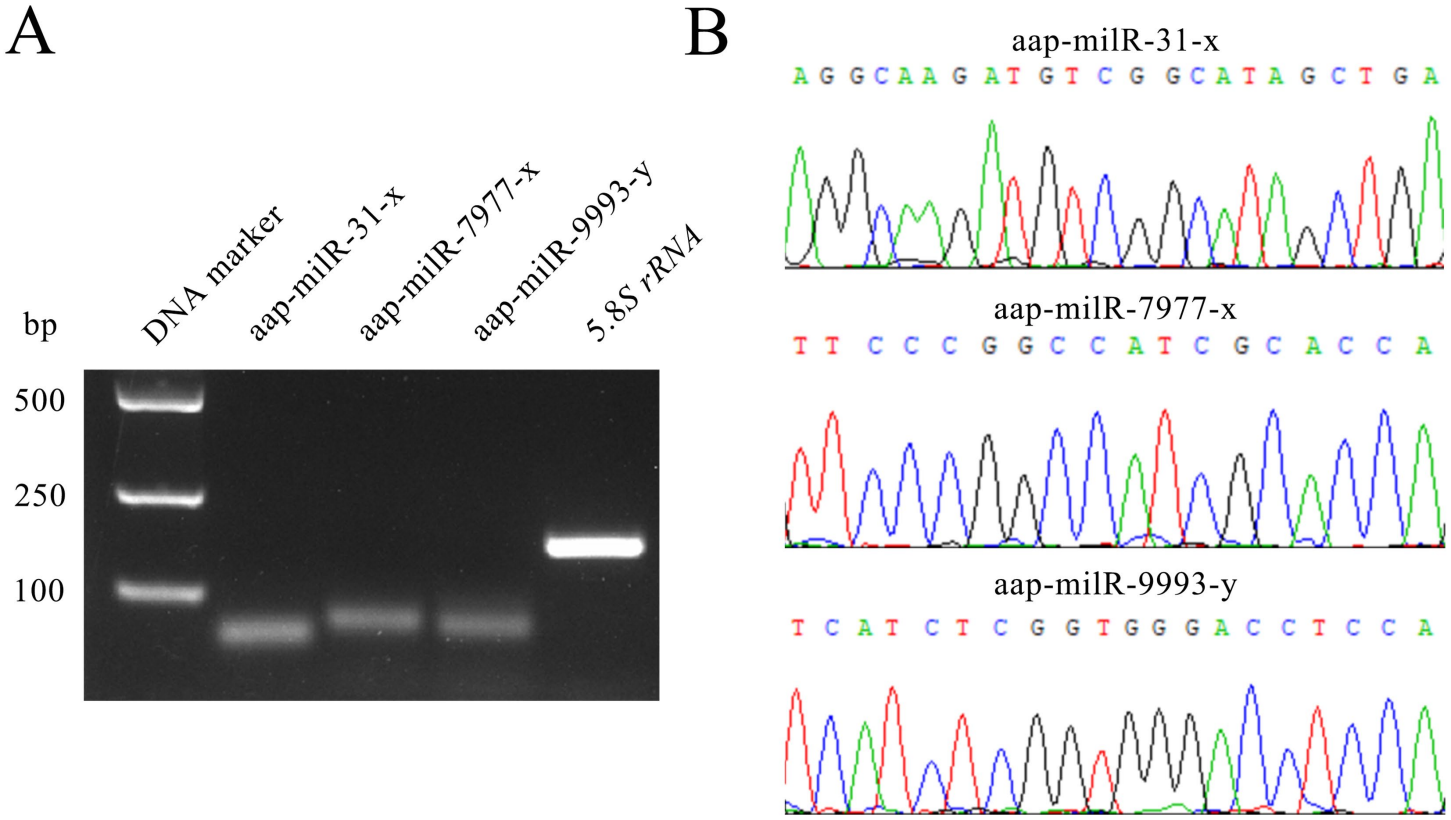
Molecular validation of expression and sequences of *A. apis* milRNAs. **(A)** Agarose gel electrophoresis for the amplification products from Stem-loop RT-PCR of aap-milR-31-x, aap-milR-7977-x, and aap-milR-9993-y; **(B)** Peak diagrams of Sanger sequencing of the amplified fragments from aap-milR-31-x, aap-milR-7977-x, and aap-milR-9993-y.

Agarose gel electrophoresis demonstrated that the fragments with expected sizes (approximately 100 bp) were amplified from aap-milR-31-x, aap-milR-7977-x, and aap-milR-9993-y (Figure 2A). Sanger sequencing suggested that the sequences of amplified fragments were consistent with those in the transcriptome sequencing data, verifying the authenticity of their sequences (Figure 2B).

**Figure 3:**
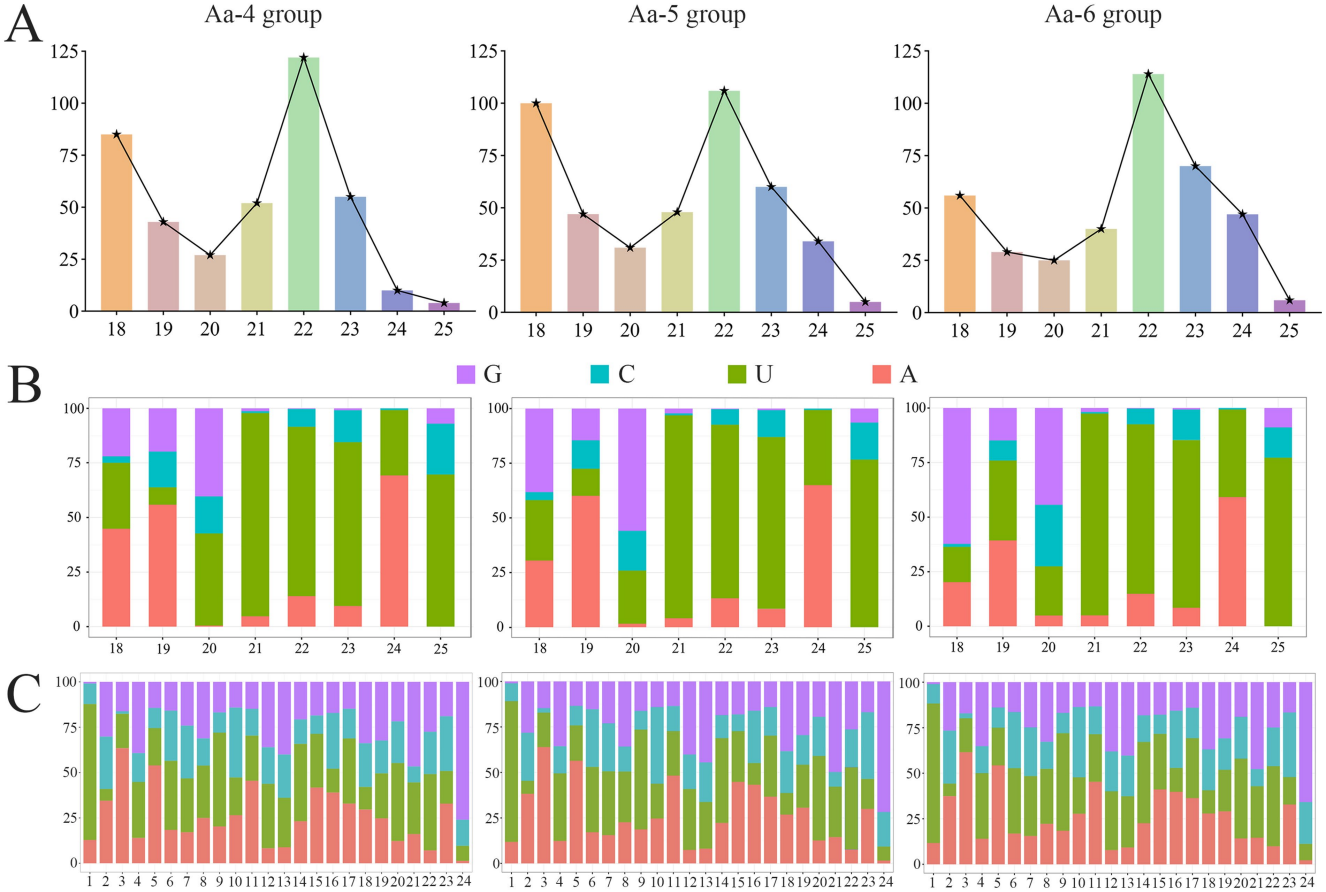
The structural property of milRNAs identified in 4-, 5-, and 6-day-old larval guts infected by *A. apis*. **(A)** Length distribution of milRNAs; **(B)** First base bias of milRNAs; **(C)** Base bias of milRNAs at each base.

In the Aa-4, Aa-5, and Aa-6 groups, aap-milR-31-x, aap-milR-7977-x, and aap-milR-9993-y were, respectively, identified, their length distribution were ranged from 18 nt to 25 nt, with the most milRNAs distributed in 22 nt (Figure 3A). In addition, the first base of milRNAs was always biased toward U (Figure 3B), and milRNAs exhibited various base biases at each base (Figure 3C).

### 3.3 Trend analysis of *Ascosphaera apis* milRNAs

**Figure 4:**
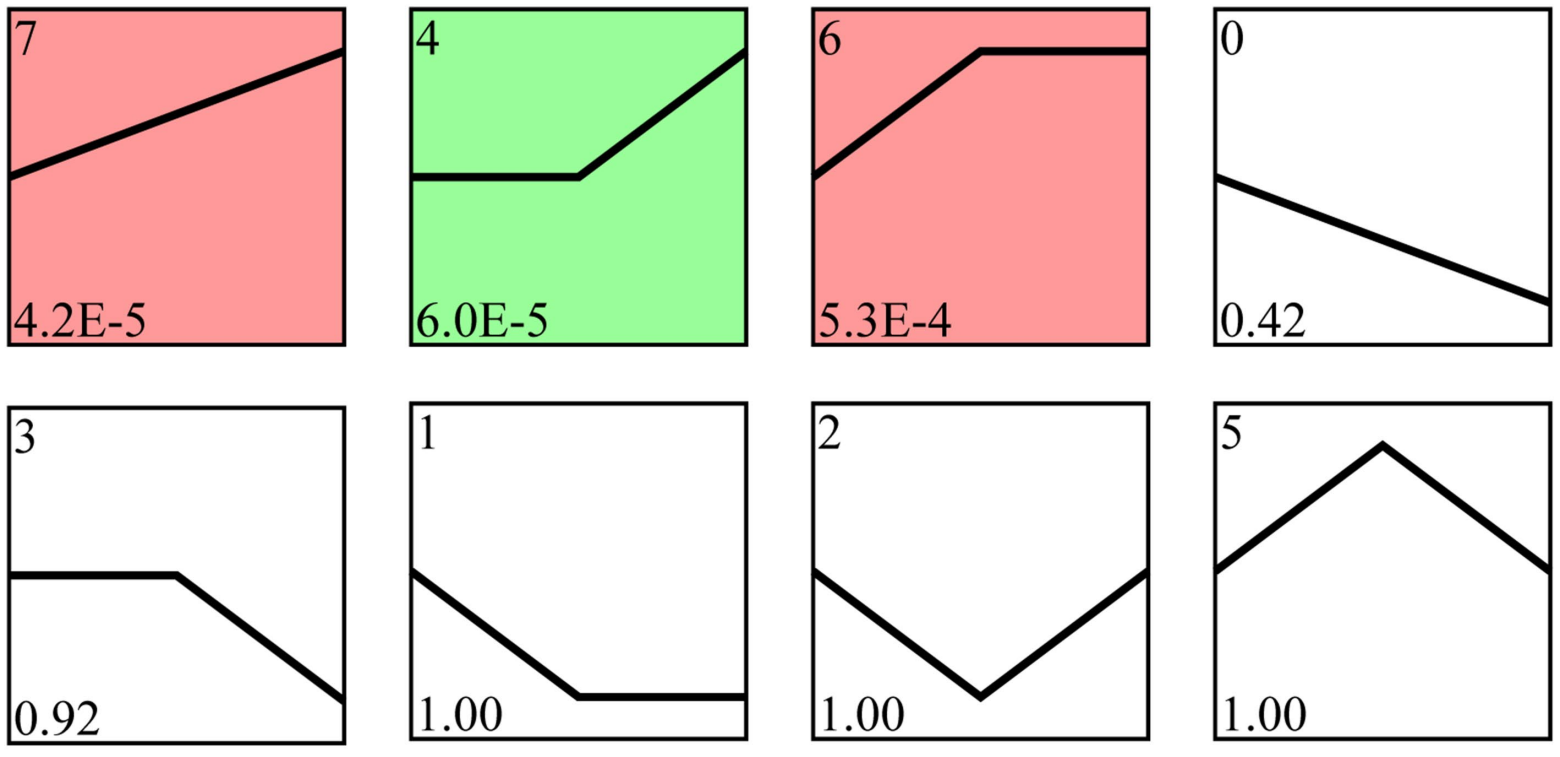
Trend analysis of *A. apis* milRNAs. Colorful squares represent significant trends, while colorless squares represent non-significant trends; the numbers located in the upper left of each square indicate different trends, whereas those located in the lower left of each square indicate the *p* value of each trend.

Following trend analysis, 113 milRNAs were divided into 8 profiles, including three significant profiles (profile4, profile6, and profile7). There were 29, 18, and 32 milRNAs included in profile4, profile6, and profile7, further targeting 4,457, 4,469, and 3,353 mRNA, respectively (Figure 4).

### 3.4 GO term and KEGG pathway annotation of mRNAs targeted by milRNAs in significant trends

**Figure 5:**
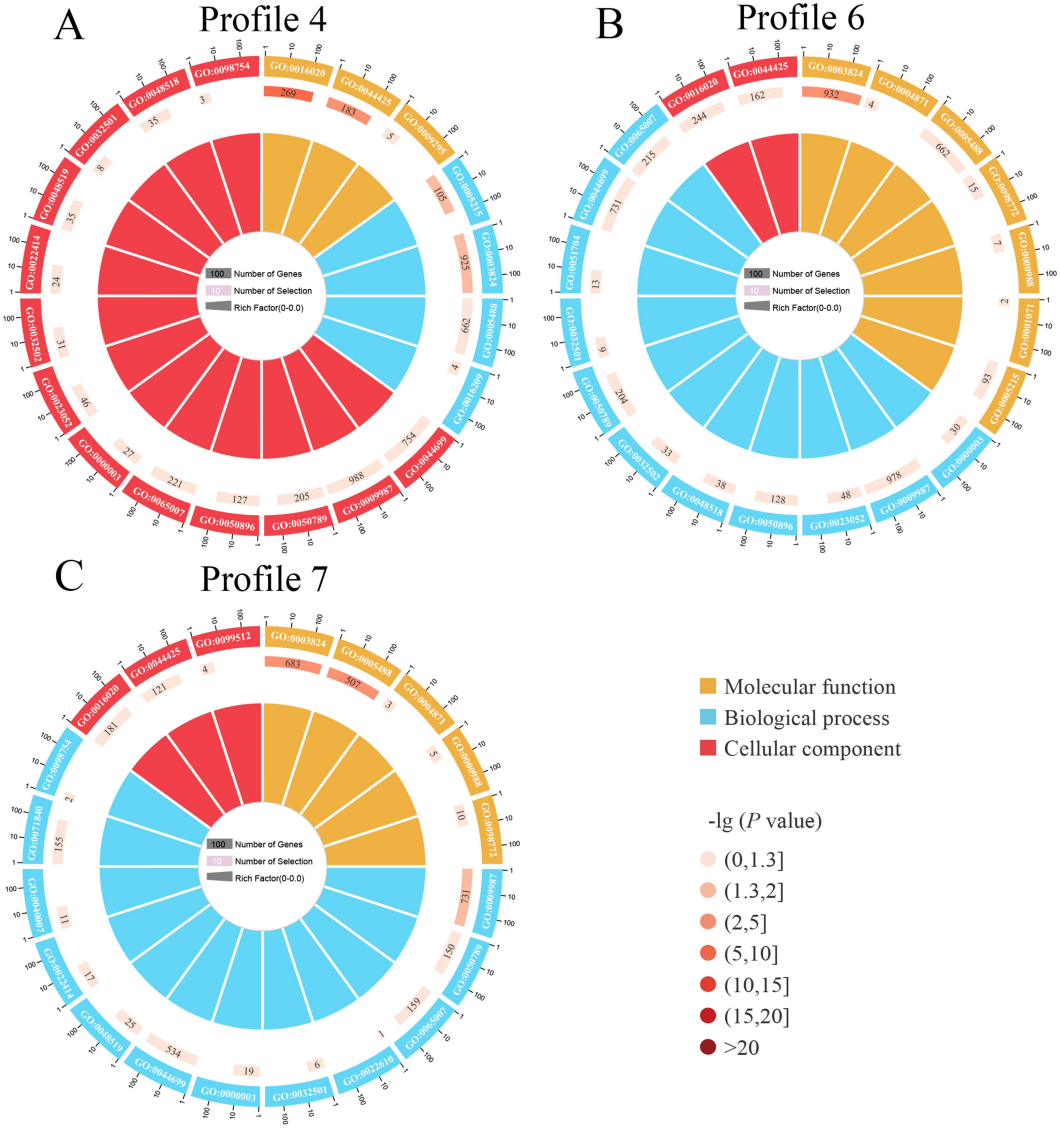
Loop diagrams of GO terms enriched by target mRNAs of *A. apis* milRNAs within 3 significant trends including profile4 **(A)**, profile6 **(B)**, and profile7 **(C)**.

It’s found that 4,457 target mRNAs in profile4 were annotated to 42 functional terms in the GO database, including 19 Biological Process terms (localization, single-organism process, and cellular process, etc.), 11 Molecular Function terms (transporter activity, catalytic activity, and binding, etc.), and 12 Cellular Component terms (membrane, cell, and cell part, etc.), the proportions of these three terms were as follows: 45.2, 26.2, and 28.6% (Figure 5, see also Supplementary Table S2); 4,469 target mRNAs in profile6 were involved in 42 functional terms including 45.2% Biological Process terms (19 types), 26.2% Molecular Function terms (11 types), and 28.6% Cellular Component terms (12 types) (Figure 5, see also Supplementary Table S3), while 3,275 targets in profile7 were associated with 41 terms including 46.3% Biological Process terms (19 types), 24.4% Molecular Function terms (10 types), and 29.3% Cellular Component terms (12 types) (Figure 5, see also Supplementary Table S4).

**Figure 6:**
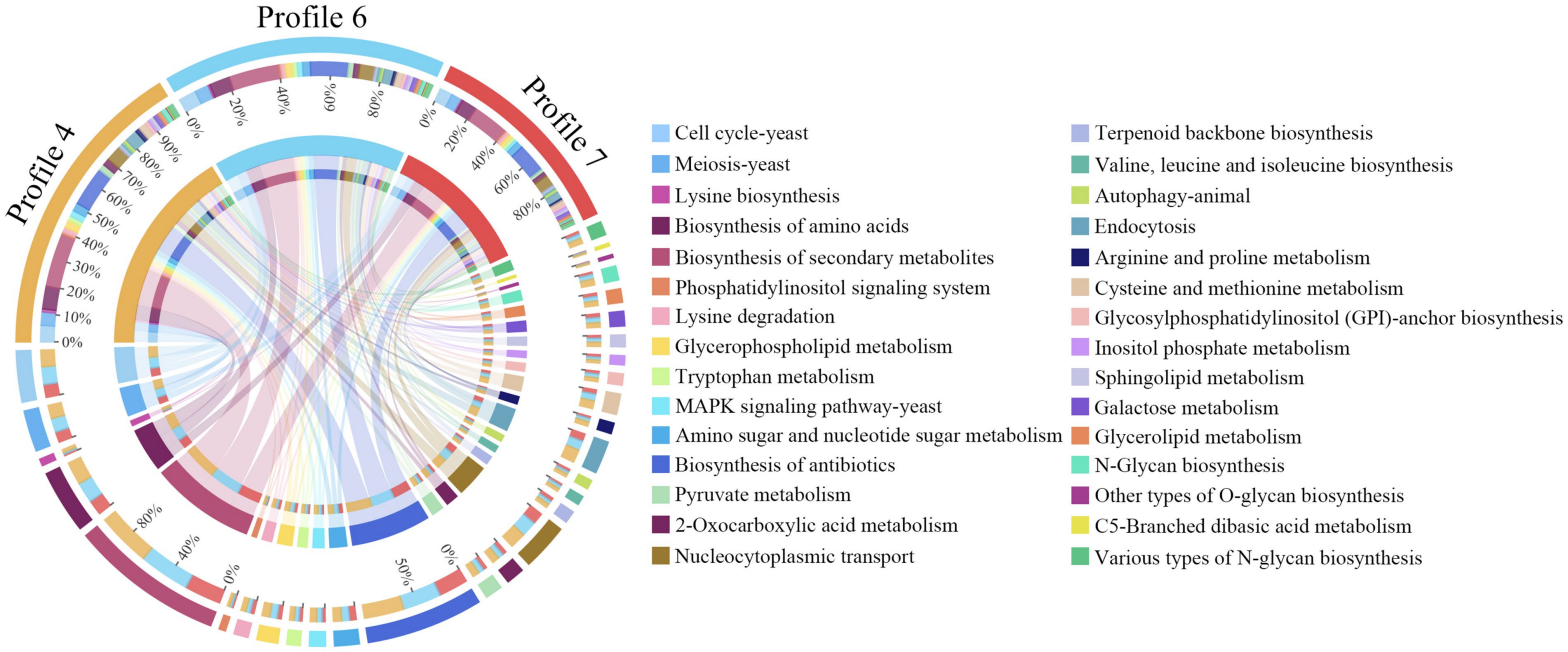
Chord diagrams of KEGG pathways enriched by *A. apis* milRNA-targeted mRNAs in 3 significant trends. The scale value represents the proportion of the corresponding color label. The line in the middle indicated the presence of pathways in a Profile, and the thicker the line segment, the more genes could be annotated to the pathway.

In contrast to GO annotation, KEGG enrichment focused on pathway-level interactions and regulation of genes in biological systems. Here, 120 KEGG pathways were enriched by 4,457 target mRNAs of DEmilRNAs in profile 4, such as lysine biosynthesis, phosphatidylinositol signaling system, and pyruvate metabolism (Figure 6, see also Supplementary Table S5), 4,469 target mRNAs of DEmilRNAs in profile 6 were involved in 119 pathways like glycosylphosphatidylinositol (GPI)-anchor biosynthesis, galactose metabolism, and endocytosis (Figure 6, see also Supplementary Table S6), whereas 3,275 target mRNAs of DEmilRNAs in profile7 were relevant to 118 pathways including biosynthesis of antibiotics, tryptophan metabolism, and sphingolipid metabolism (Figure 6, see also Supplementary Table S7).

### 3.5 Regulatory networks between DEmilRNA in significant trends and target mRNAs

**Figure 7:**
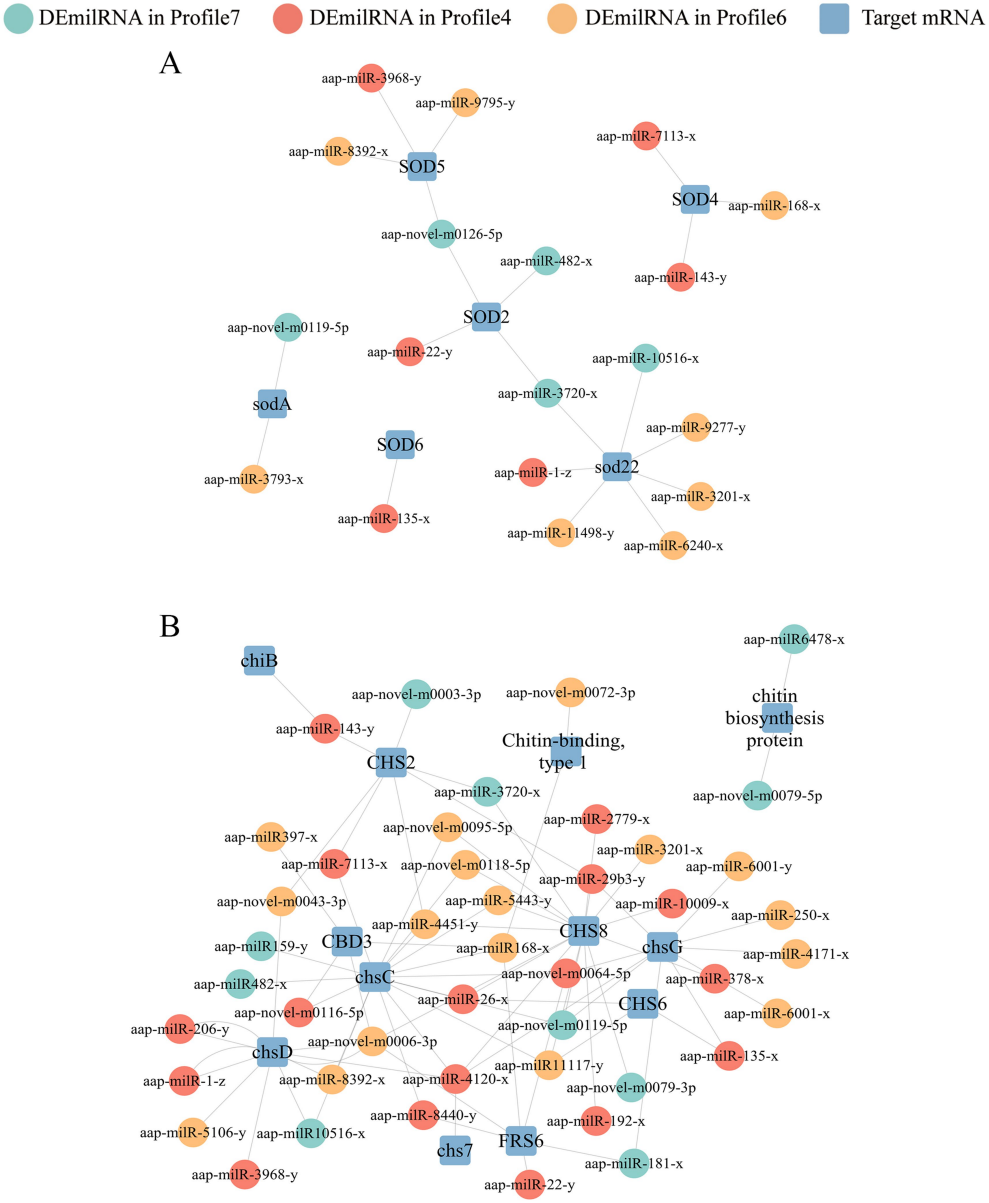
Regulatory networks between *A. apis* DEmilRNAs in significant trends and target mRNAs associated with virulence factors. **(A)** Regulatory network of milRNAs-targeted superoxide dismutase-encoding mRNAs; **(B)** Regulatory network of milRNAs-targeted enzymes related to chitin synthesis and degradation-encoding mRNAs.

It’s detected that 25, 27, and 15 DEmilRNAs in profile 4, profile 6, and profile 7 could target 25 mRNAs (*mkh*1, *rhoA*, and *hog*1, etc.) related to the MAPK signaling pathway (Supplementary Figure S2). Specifically, 16 DEmilRNAs in profile 4, 19 DEmilRNAs in profile 6, and 11 DEmilRNAs in profile 7 could target 21 glycerolipid metabolism-relevant mRNAs such as *aglA*, *aglD*, and *gar*1 (Supplementary Figure S3). A total of 63 DEmilRNAs could target 18 virulence factor-relevant mRNAs (Figure 7). In detail, 6, 8, and 5 milRNAs were observed to target 6 superoxide dismutase-encoding mRNAs, including Na^+^/H^+^ antiporter Nha1 (*SOD*22), Cation/H^+^ exchanger (*SOD*2), and Cu, Zn superoxide dismutase SOD1 (*sodA*) (Figure 7A). Additionally, 17, 17, and 10 milRNAs were found to target 12 mRNAs related to chitin synthesis and degradation in profile 4, profile 6, and profile 7, such as chitin synthase class VI (*chsD*), chitin synthase (*chsC*), and endochitinase 1 (*chiB*) (Figure 7B). Furthermore, it’s noticed that several milRNAs, such as aap-milR-3720-x, aap-milR-4120-x, and aap-milR-4451-y, can target more than 1 mRNA (Figure 7).

### 3.6 Verification of binding relationships between the *Ascosphaera apis* DEmilRNAs and target genes

**Figure 8:**
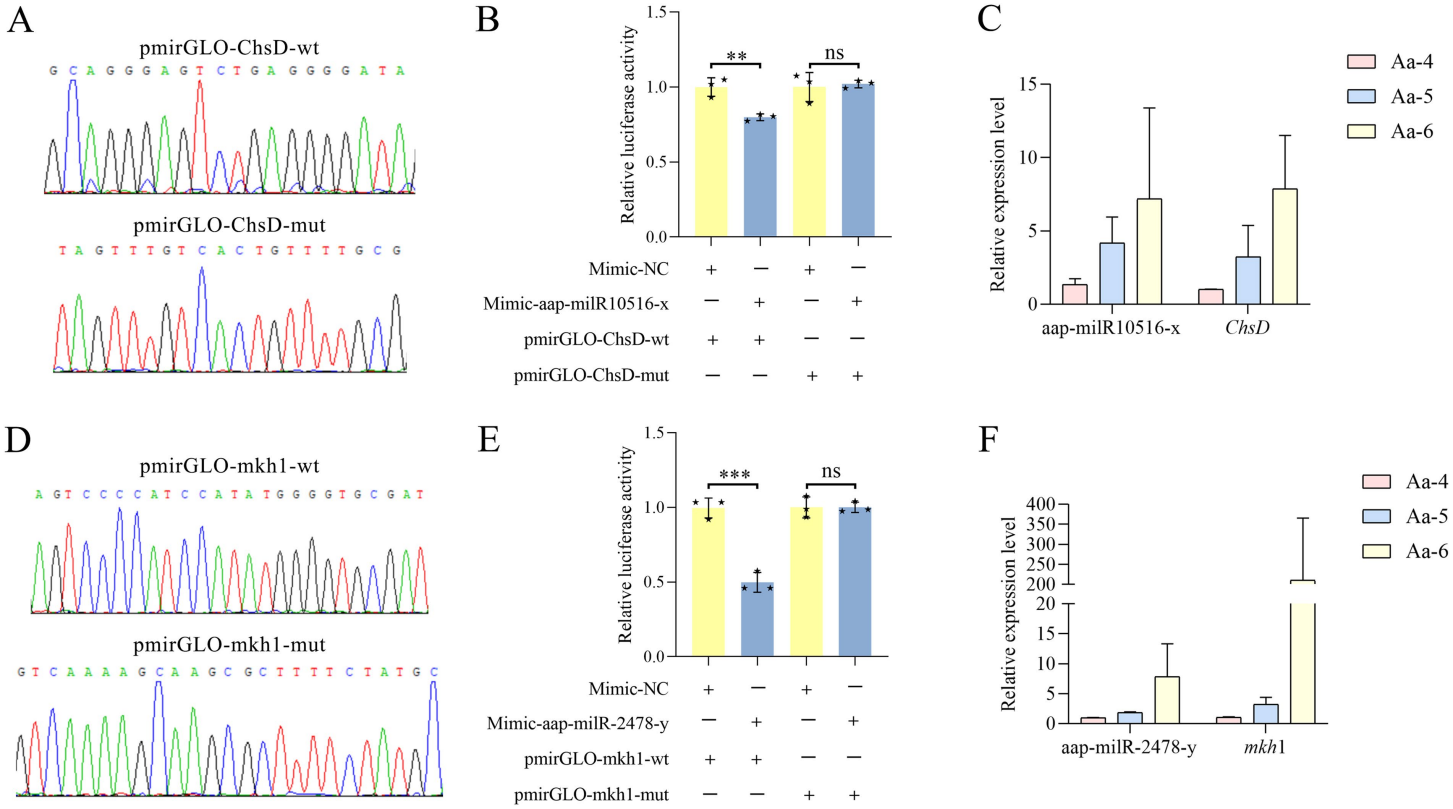
Validation of binding relationships between *ChsD* and aap-milR10516-x and between *mkh*1 and aap-milR-2478-y. **(A)** Peak diagram of Sanger sequencing of the amplified binding sites. **(B)** Dual-luciferase reporter gene assay of the binding relationship between aap-milR10516-x and *ChsD*. **(C)** RT-qPCR detection of aap-milR10516-x and *ChsD*. **(D)** Peak diagram of Sanger sequencing of the mutated binding sites. **(E)** Dual-luciferase reporter gene assay binding relationship between aap-milR-2478-y and *mkh*1. **(F)** RT-qPCR detection of aap-milR-2478-y and *mkh*1. The dual-luciferase assay data were presented as mean ± standard deviation (SD) and analyzed by two-sided Student’s *t*-test; ns, *p* > 0.05; **p < 0.01; ***p < 0.001.

Here, recombinant plasmids pmirGLO-ChsD/mkh1-wt and pmirGLO-ChsD/mkh1-mut were successfully constructed, as shown in Figures 8A,D. Dual-luciferase reporter gene assay suggested that the luciferase activities in the wt co-transfection groups were significantly decreased as compared to that in the corresponding control groups; comparatively, in both two mut co-transfection groups, the luciferase activities were non-significantly different in comparison with those in the corresponding mut control group (Figures 8B,E). These results together were indicative of the binding relationships between aap-milR10516-x and *ChsD* as well as between aap-milR-2478-y and *mkh*1.

RT-qPCR results demonstrated that in the 4-, 5-, and 6-day-old larval guts infected by *A. apis* the expression level between aap-milR-2478-y and *mkh*1 was positively correlated, and a similar relationship was also observed between aap-milR10516-x and *ChsD* (Figures 8C,F).

## 4 Discussion

To facilitate infection, pathogens typically deliver biomacromolecules such as effector proteins, nucleic acids, and toxins into the host. In recent years, it has been demonstrated that milRNAs can participate in regulating fungal spore formation (Zhou et al., 2012; Zeng et al., 2018; Zhang E. et al., 2022; Zhang W. D. et al., 2022). Zhang et al. reported that milRNAs can influence the morphology and structure of *Metarhizium acridum* spores (Zhang E. et al., 2022; Zhang W. D. et al., 2022). *A. apis* specifically infects honeybee larvae and has developed specific infection patterns through long-term coevolution with its host (Li et al., 2012). However, current understanding of the pathogenic mechanisms of *A. apis* is limited to histological and biochemical levels, lacking in-depth elucidation at the molecular level. In this work, we for the first time conducted a comprehensive analysis and exploration of milRNAs in *A. apis* spores during the infection process, including their abundance, structural features, expression profiles, target mRNAs, and regulatory networks, based on high-quality sRNA-seq data. This study provides a foundation for understanding the mechanisms of *A. apis* infection in the Asian honeybee larvae mediated by milRNAs and offers a new perspective for the prevention and control of chalkbrood disease in honeybees.

Here, we identified a total of 398, 431, and 387 milRNAs ranging in length from 18 to 25 nt, with the highest number of milRNAs found at a length of 22 nt (Figures 3A,B). The first nucleotide of these milRNAs is predominantly U (Figure 3C), and their structural features are highly similar to milRNAs discovered in various fungi such as *Coprinopsis cinerea* (Lau et al., 2018), *Curvularia lunata* (Liu et al., 2016), *Penicillium marneffei* (Lau et al., 2013), as well as in animals and plants like honeybees and cotton (Li et al., 2019; Xiong et al., 2018). The 253 shared milRNAs among the three groups exhibited varying expression patterns during the infection process of *A. apis* (Figure 1C). It’s inferred that these common milRNAs are likely to play essential roles during the whole process of *A. apis* infection, hence deserving more attention and further investigation.

In recent years, utilizing STEM software for trend analysis has been widely applied in diverse species, such as *Columba livia* (Wang et al., 2020), *Bos grunniens* (Liu et al., 2023), *Solenopsis Invicta* (Wu et al., 2023), and *Chilo suppressalis* (Bao et al., 2022). Wu et al. (2023) analyzed the DEGs of *Metarhizium anisopliae* during *S. invicta* infection by using STEM software and discovered that *M. Anisopliae* inhibited the immune response of *S. invicta* in the early stage but activated the host immune response in the later stage. Based on the trend analysis, Wang et al. (2020) analyzed the DEmiRNAs in the liver of *C. livia* at different developmental stages and found these DEmiRNAs with different expression patterns through enrichment analysis of target mRNAs might play critical roles in response to growth factors, cellular morphogenesis, and glandular development. In this current work, trend analysis revealed that 79 milRNAs in *A. apis* invading the Asian honeybee worker larvae could be classified into three significant trends: Profile4, Profile6, and Profile7 (Figure 4). The GO term analysis of their target mRNAs mainly included functional terms such as localization, antioxidant activity, and nucleoid (Figure 5). Enriched KEGG pathways primarily included lysine biosynthesis, pyruvate metabolism, and biosynthesis of antibiotics (Figure 6). These targets were implicated in pathogen activation.

In fungi, glycerolipid metabolism is involved in lots of biological processes, such as cell division and growth, energy metabolism, and membrane biogenesis (Henry et al., 2014; Henry et al., 2012). *Nosema ceranae*, another widespread fungal disease of honey bees, has been shown to cause energy stress on the host (Mayack and Naug, 2009). Here, the results showing 11, 16, and 19 DEmilRNAs in Profile4, Profile6, and Profile7, respectively, could target 21 glycerolipid metabolism-encoding mRNAs (Supplementary Figure S3), which suggested that these DEmilRNAs possibly regulated the energy metabolism through their related target mRNAs.

Moreover, a total of 67 DEmilRNAs were detected to target 25 mRNAs engaged in the MAPK signaling pathway (Supplementary Figure S2). After being stimulated by external environmental factors, pathogenic fungi can initiate complex and conserved signal transduction cascades, leading to changes in the expression of downstream target mRNAs (Lange et al., 1999). The MAPK signaling pathway plays a crucial role in modulating various biological processes in fungi, including spore formation (Zhao et al., 2007), virulence levels (Igbaria et al., 2008), reproduction (Chen et al., 2016), and pathogenicity (Rodríguez-Peña et al., 2010). Chen et al. (2016) investigated the complete complementarity of the MAPK cascade components in *Metarhizium robertsii* and found that the Hog1 and Slt2-MAPK cascades contributed to pathogenicity, and the Fus3-MAPK cascade was indispensable for the fungal pathogenesis. Chen et al. (2017) observed that 48 DEGs in *A. apis* were enriched in the MAPK signaling pathway during the infection of *A. m. ligustica* larvae, and their expression levels significantly increased with prolonged fungal pathogen stress. However, for the *A. apis* infecting *A. c. cerana* larvae, Guo et al. (2017) found that 11 DEGs enriched in the MAPK signaling pathway displayed a downward trend. Here, 15, 25, and 27 DEmilRNAs (eg. aap-milR-3720-x, aap-milR-1-z, and aap-milR-6001-y) were observed to bind to 25 target mRNAs associated with the MAPK signaling pathway of *A. apis* in profile7, profile4, and profile6, respectively (Supplementary Figure S2). It speculated that these DEmilRNAs of *A. apis* may act as pathogenic factors involved in regulating the MAPK signaling pathway of *A. apis*, and participated in the process of *A. apis* infecting the *A. cerana* worker larvae.

During the infection of honeybee larvae by *A. apis* in the gut, the pathogen is capable of secreting various enzymes such as chitinases, proteases, lipases, and secondary metabolites, which act as virulence factors to influence and bypass the host’s immune defense system (Aronstein and Murray, 2010). Chitin synthase was an important virulence factor of entomopathogenic fungi, participating in the synthesis of fungal cell walls and play a key part in maintaining fungal cell morphology, stress resistance, pathogenicity, and other aspects (Fiorin et al., 2018). It also cooperated with other virulence factors such as proteases, then degraded the insect’s peritrophic membrane and cuticle (Leger, 1996). In the present study, 10, 17, and 17 DEmilRNAs from profile7, profile4, and profile6 could target enzymes related to chitin synthesis and degradation gene-encoding mRNAs, such as aap-milR-3968-y, aap-milR-1-z and aap-milR-3201-x (Figure 7B). This suggested that these DEmiRNAs could affect the pathogenesis of *A. apis* through regulating the expression of enzymes related to chitin synthesis and degradation genes. Superoxide dismutase (SOD) were a ubiquitous family of enzymes widely discovered in animals (Miao and St Clair, 2009), plants (Gill et al., 2015), and microorganisms (Brioukhanov and Netrusov, 2004). Previous studies have demonstrated that SOD in fungi could affect virulence (Papapostolou and Georgiou, 2010; Fréalle et al., 2005). Martchenko et al. (2004) reported that SOD5 was a virulence factor for *Candida albicans* and was important for fungal survival against the oxidative attack of macrophages and neutrophils. Here, 6 dismutase gene-encoding mRNAs were detected to be targeted by 5, 6, and 8 DEmilRNAs in profile7, profile4, and profile6 (Figure 7A). This indicated that the relevant DEmilRNAs could affect the virulence of *A. apis* the modulation of their target mRNAs. *ChsD* is one of the fungal chitin synthase genes and play an important role in maintaining fungal morphology. Chitin synthesized by the *ChsD*-encoded isozyme contributes to the rigidity of the walls of germinating conidia, the rigidity of hyphae subapical region and conidiophore vesicles (Specht et al., 1996). *mkh*1 is an important gene involved in the MAPK signaling pathway. The positive regulatory relationship between milR10516-x and *ChsD* as well as between milR-2478-y and *mkh*1 were validated through RT-qPCR and dual-luciferase reporter gene assays. miRNAs achieve degradation or translation inhibition primarily by targeting the 3’UTR of mRNA transcripts in the cytoplasm. The miRNAs are still considered as repressors of gene expression in the mainstream view. However, there is growing evidence of non-canonical novel role in the nucleus of miRNAs, acting as activators or silencing of target gene transcription through miRNA-promoter interaction (Sarkar and Kumar, 2025). Studies have shown that mir-23a and miR-23b directly target *Hmgn2*, promoting transcriptional activation at several gene promoters, including the *amelogenin* gene promoter (Eliason et al., 2022). miR-24-1 activates gene transcription by targeting enhancers. When the enhancer sequence is removed, the activation is completely eliminated. In addition, miR-24-1 activates enhancer RNA expression, alters histone modification, and increases enrichment of p300 and RNA Pol II at the enhancer locus (Xiao et al., 2017). In our past studies of another fungal pathogen *Nosema ceranae*, we found that the structure of *N. ceanae* mRNAs were very simple, and almost all mRNAs lack a 3’UTR structure (Zhang E. et al., 2022; Zhang W. D. et al., 2022). Similarly, as a microorganism, *A. apis* has a very simple structure, perhaps this is what causes *A. apis* milRNAs to positively regulate genes. This not only proved the authenticity of sequencing data, but also provided two candidate DEmilRNA/mRNA axes for further in-depth research on the function of milRNA during the infection of *A. cerana* by *A. apis*.

## 5 Conclusion

**Figure 9:**
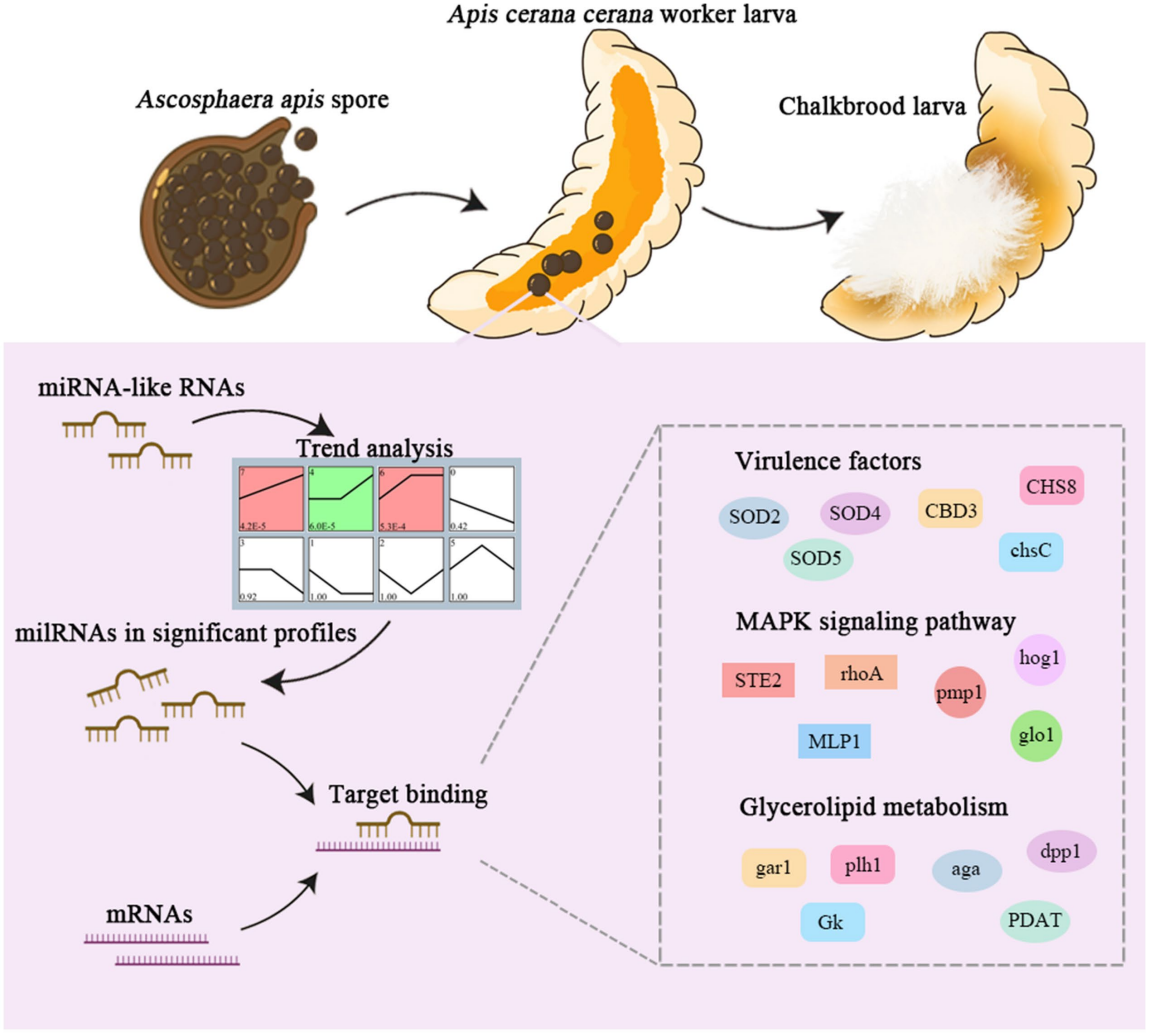
A hypothetical working model of milRNA regulation in *A. apis* infecting the *A. c. cerana* larvae.

In conclusion, a total of 606 *A. apis* milRNAs were discovered, with lengths ranging from 18 nt to 25 nt. Among these, 253 milRNAs were shared by *A. apis* infecting 4-, 5-, and 6-day-old larvae (Figure 1B). These common milRNAs exhibited different expression patterns during the infection process, with 68 milRNAs upregulated and 54 ones downregulated (Figure 1C). Furthermore, the expression of three milRNAs was validated. 79 milRNAs of *A. apis* could be classified into three significant trends (Profile4, Profile6, and Profile7), and these DEmilRNAs mainly targeted mRNAs associated with glycerol metabolism, mRNAs in the MAPK signaling pathway, and mRNAs encoding virulence factors like enzymes related to chitin synthesis and degradation and superoxide dismutase. The positive regulatory relationship between milR10516-x and *ChsD* as well as between milR-2478-y and *mkh*1 were verified (Figure 8). These findings not only lay a foundation for understanding the mechanism of *A. apis* infection in the Asian honeybee larvae mediated by milRNAs, but also offer new insights into the prevention and control of chalkbrood disease in honeybees (Figure 9).

## Data Availability

Raw data generated from RNA-seq are available in the NCBI SRA database under the BioProject number PRJNA565611.

## Footnotes

^1^ https://www.ncbi.nlm.nih.gov/genbank/

^2^ http://rfam.xfam.org/

^3^ http://www.ncbi.nlm.nih.gov/Web/Genbank/

^4^ http://rfam.xfam.org/

^5^ http://oberon.fvms.ugent.be:8080/rRNA/lsu/index.html

^6^ https://www.mirbase.org/

^7^ http://www.geneontology.org/

^8^ https://www.kegg.jp/

^9^ http://www.omicshare.com/tools/index.php/
