# Supplementary figures and images for: Global discovery, expression pattern, and regulatory role of miRNA-like RNAs in Ascosphaera apis infecting the Asian honeybee larvae
Source: Front Microbiol. 2025 Mar 4;16:1551625. doi: 10.3389/fmicb.2025.1551625 (PMC11914139; doi:10.3389/fmicb.2025.1551625)

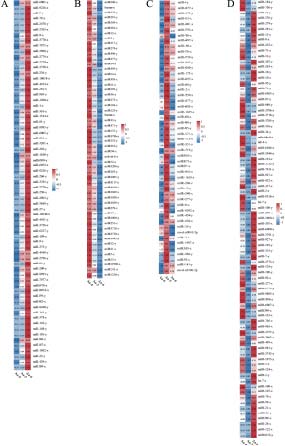

Supplement: Supplementary file 1 [file Image_1.jpeg]

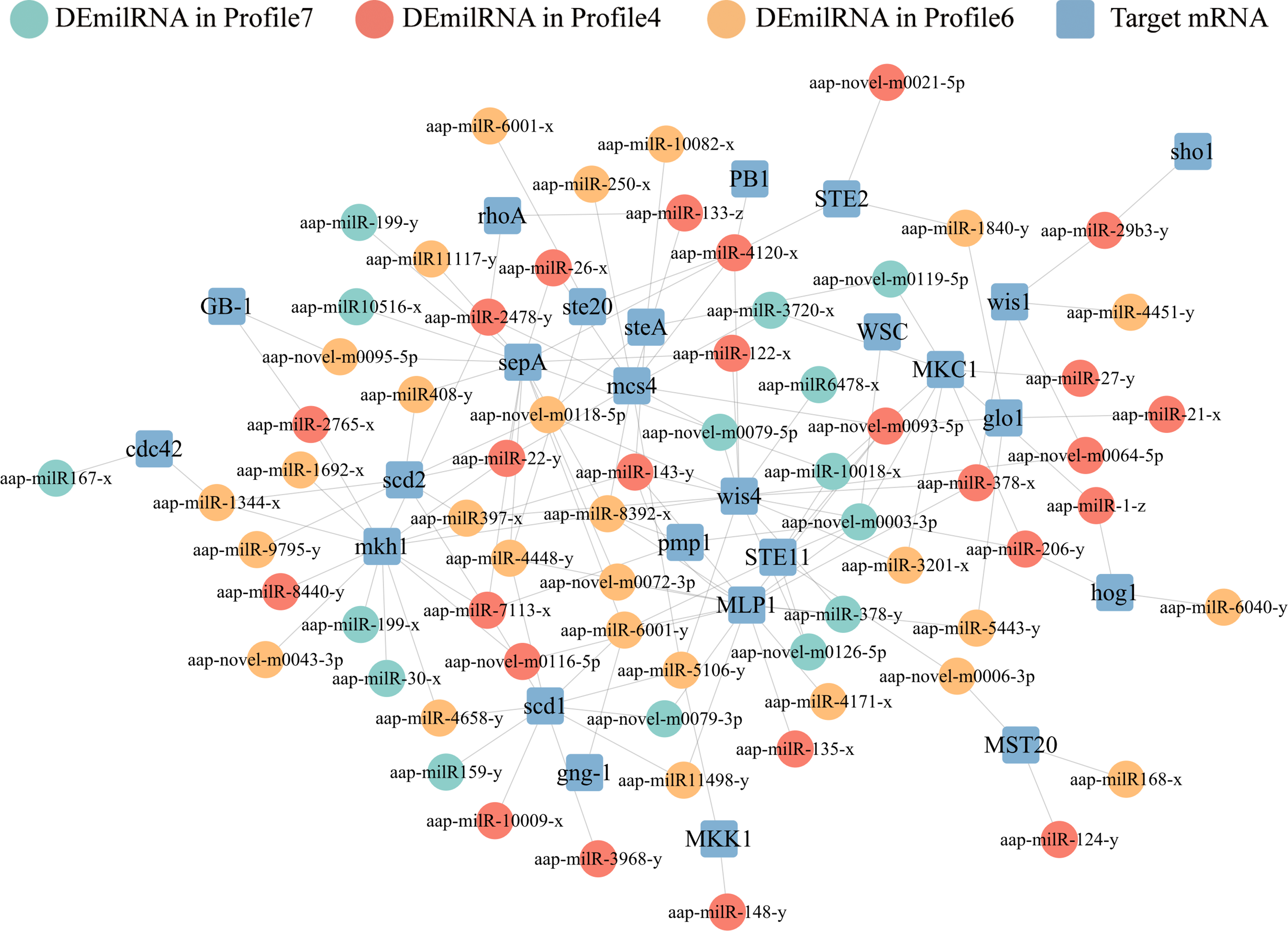

Supplement: Supplementary file 2 [file Image_2.tif]

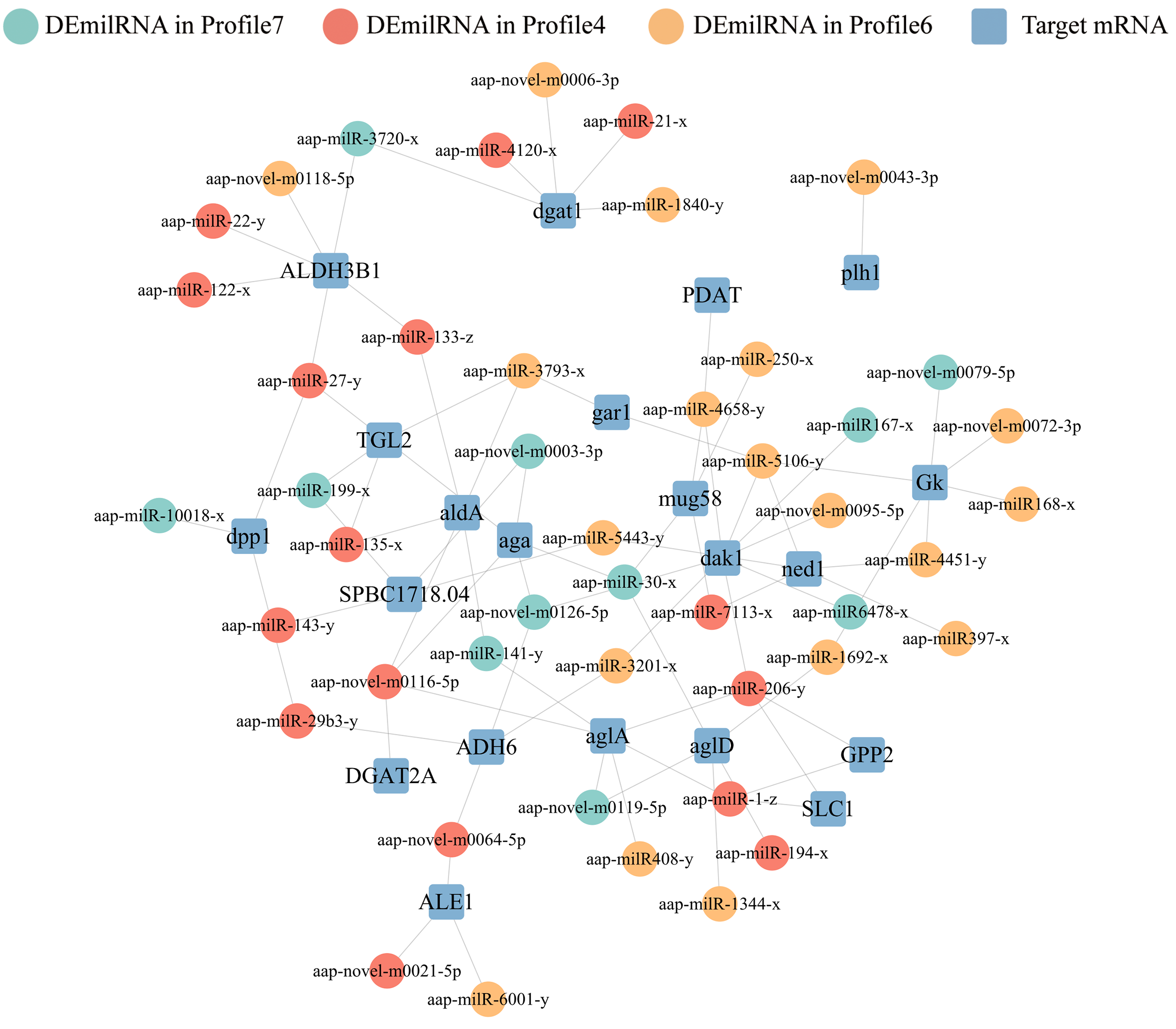

Supplement: Supplementary file 3 [file Image_3.TIF]
